# From Diagnosis to Fertility: Optimizing Treatment of Adenomyosis for Reproductive Health

**DOI:** 10.3390/jcm13164926

**Published:** 2024-08-21

**Authors:** Hanna Kim, Emily H. Frisch, Tommaso Falcone

**Affiliations:** Women’s Health Institute, Cleveland Clinic Foundation, Cleveland, OH 44195, USA; kimh14@ccf.org (H.K.); frische@ccf.org (E.H.F.)

**Keywords:** adenomyosis, surgical management options, fertility preservation

## Abstract

Adenomyosis is a benign gynecologic disorder that had previously not been well studied or understood. However, it is now become a more common diagnosis with long-standing implications especially for fertility. In this literature review, the pathophysiology and diagnosis along with management options for uterine preservation and fertility along with more definitive options are reviewed. While there is a better understanding of adenomyosis, there is still more research that is needed to fully elucidate the best ways of management for patients especially in those seeking fertility.

## 1. Introduction

Adenomyosis is a benign disorder in which endometrial tissue implants within the uterine myometrium. In the past, there was no differentiation between endometriosis and adenomyosis. The active definition that was originally used for endometriosis which ultimately came to be known as adenomyosis was defined as “benign invasion of endometrium into the myometrium, producing a diffusely enlarged uterus” [1]. This ectopic endometrial tissue induces hypertrophy and hyperplasia of the surrounding smooth muscle myometrial cells resulting in a diffusely enlarged uterus [2]. Recently, there has been more of a shift to identify the two as different disorders, although commonly occurring simultaneously in one patient. This observation has led to the concept of some unifying or overlapping pathway for both disorders. With additional research into adenomyosis, there has been more understanding of gynecologic issues associated with adenomyosis. Adenomyosis involves the myometrial junctional zone, which is crucial for spiral artery remodeling and alteration of the vascularity of the myometrium, potentially resulting in impaired decidualization leading to obstetric and fertility complications [3].

## 2. Diagnosis of Adenomyosis

The clinical presentation of adenomyosis is often mixed, and occasionally, some may even be asymptomatic [4]. The symptoms related to adenomyosis include menorrhagia, pelvic pain, and dysmenorrhea, which can lead to decreased quality of life [5]. The two pathological components of adenomyosis are diffuse and focal forms (when a defined nodule is found, the term adenoma is also used). Diffuse adenomyosis leads to more severe menstrual-related symptoms [6], and symptoms experienced by women with diffuse adenomyosis are more pronounced than those with focal adenomyosis [3].

With recent advances in imaging technology, adenomyosis can now be accurately diagnosed via ultrasound (US), magnetic resonance imaging (MRI), as well as pathological report of excised tissue [7]. Transvaginal ultrasound is preferred for diagnosis of adenomyosis. The ultrasound criteria of adenomyosis include heterogeneous myometrial area, globular asymmetric uterus, irregular cystic spaces, myometrial linear striations, poor definition of endometrial myometrial junction, and myometrial anterior posterior asymmetry with thickening of anterior and posterior myometrial wall and increased or decreased echogenicity. A practical ultrasound classification of adenomyosis is given in Table 1, originally published by Lazzeri et al. 2018 [8]. Currently, the features of the Morphological Uterus Sonographic Assessment (MUSA) are diagnostic for adenomyosis. These features include direct features such as myometrial cysts, hyperechogenic myometrial islands, and echogenic sub-endometrial lines and buds along with indirect features such as asymmetrical thickening, fan-shaped shadowing, globular uterus, irregular or interrupted junctional zone, and translesional vascularity [9,10]. As seen in the table below, the description and location of adenomyosis yields a classification score of 1 through 4 and can help categorize the lesions noted [8]. The presence of adenomyoma is suggested by the presence of nonhomogeneous circumscribed areas in the myometrium with indistinct margins seen on ultrasound and can be another sign of more global disease. Another criteria that could be helpful for diagnosis include the question-mark-form sign, which occurs when the uterine corpus is flexed backwards with the cervix directed anteriorly and the uterus facing the posterior pelvis compartment which makes the endometrium create a “question mark” sign [11]. Specificity and sensitivity for the question mark sign was 93% and 75% [11].

**Figure 1:**
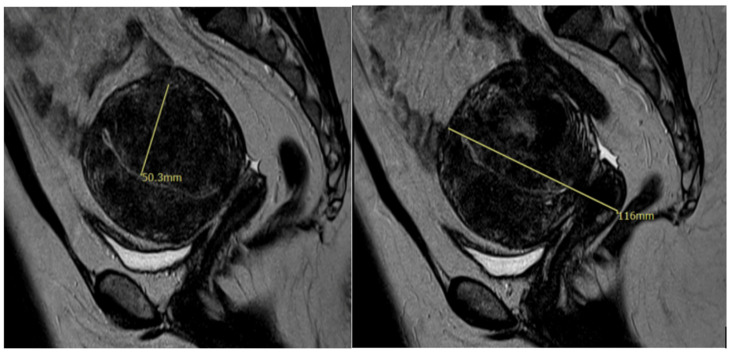
MRI image showing diffuse adenomyosis.

Additionally, MRI has become an important diagnostic tool for adenomyosis. MRI criteria of adenomyosis include a myometrial mass with indistinct margins of primarily low intensity, diffuse or local widening of junctional zones on T2-weighted images with thickness > 12 mm, uterine enlargement, and small hypointense myometrial spots [12]. An MRI representation of an adenomyoma is shown in Figure 1. While characteristics of adenomyosis on ultrasound likely represent a true diagnosis, a study has shown that the rate of diagnosis is low. Among symptomatic women undergoing a pelvic ultrasound in a general gynecology clinic, 21% had a diagnosis of adenomyosis through imaging [13]. Given this, pathologic examination of the uterus postoperatively is still considered the gold standard for the diagnosis of adenomyosis.

Adenomyosis can often be found concurrently with other benign uterine pathologies [14]. Adenomyosis has been shown to coexist with other gynecological diseases such as endometriosis (20–80%) and uterine fibroids (15–57%) [5,15,16,17]. Endometrial pathology such as endometrial polyps and hyperplasia were found in 7–10% concurrently with adenomyosis [18,19]. Fibroids were found in 23–34% of patients with adenomyosis [13]. For this reason, those with adenomyosis have high health care utilization: 82.0% of women with adenomyosis had hysterectomies, nearly 70% had imaging studies suggestive of adenomyosis, and 37.6% used chronic pain medications [17].

With advances in technology, artificial intelligence (AI) and application to diagnosis of adenomyosis has been considered. In a recent publication, the role of AI in aiding in timely diagnoses was acknowledged along with understanding the subjective nature of diagnoses of adenomyosis at this time; however, there are hesitations given the inability to assess superficial and local lesions along with the inability to personalize care to the patient [20]. Additionally, in a recent study conducted looking at deep learning models in ultrasound diagnoses of adenomyosis compared to intermediate ultrasound-skilled trainees, accuracy was noted to be lower in the AI model whereas specificity was noted to be higher [21]. While there may be benefit into utilizing AI to aid with diagnoses of adenomyosis, there needs to be more understanding of exact algorithms for treatments and a consensus about efficacy of AI technology is needed so that we are able to fully utilize this technology in the treatment of patients [20].

## 3. Prevalence of Adenomyosis

The overall prevalence of adenomyosis in the general population in 2015 was 0.8%, with a larger proportion of patients noted to be on either end of the spectrum of reproductive age: 1.5% in women aged 41–45 and 34.0% in women over age 50 [17]. Incidence data vary in the literature; however, a key retrospective cohort study analyzed the incidence of symptomatic adenomyosis in over 300,000 women and found the overall adenomyosis incidence in the United States was 1.03% or 28.9 per 10,000 woman-years [17]. Incidence was noted to be highest for women aged 41–45 years (69.1 per 10,000 woman-years in 2008). The prevalence of adenomyosis on hysterectomy pathology range from 15 to 57% [18,22,23]. In adolescents with heavy menstrual bleeding or dysmenorrhea, the incidence of adenomyosis according to MUSA criteria as described in the prior section was noted to be 27.4% [24]. In a study that looked at the need for postpartum hysterectomies, adenomyosis was found in 40% of patients [25]. Additionally, studies have reported various racial differences in the incidence of adenomyosis. Incidence rates were shown to be disproportionately higher among black women: black women (highest 44.6 per 10,000 woman-years in 2011) vs. white women (highest 27.9 per 10,000 woman-years in 2010). In addition, it has been shown that non-white women are less likely to undergo laparoscopic hysterectomy compared to open hysterectomy or other treatments for adenomyosis, and non-Hispanic black women experience more major complications after hysterectomy for adenomyosis than any other race or ethnicity [26].

Because there has been difficulty in understanding the exact incidence of adenomyosis from the wide ranges reported in the literature, the direct relations between adenomyosis and infertility has been difficult to distinguish due to the coexistence of other conditions. The prevalence of adenomyosis was estimated to be 25% in women with recurrent miscarriage or repeat implantation failure undergoing assisted reproductive technology (ART) [27]. Additionally, the prevalence of adenomyosis in endometrial cancer patients was found to be 22.6% (95% confidence interval: 12.7–37.1%) in a systematic review, but the significance of this association is not yet fully understood [28]. Endometriosis has been known to negatively impact infertility and is a common concurrent finding in patients with adenomyosis [27]. However, independent of endometriosis, adenomyosis has been shown to be associated with a decrease in the cumulative live birth rate. Understanding the mechanisms underlying adenomyosis development and addressing its impact on fertility outcomes is now crucial, and a growing number of studies aim at addressing this relationship.

## 4. Proposed Pathophysiology of Adenomyosis

It has been thought that adenomyosis is caused by an imbalance in hormonal signaling between estrogen and progesterone [29]. In a mouse model that looked at adenomyosis, mice displayed disrupted estrous cyclicity, characterized by irregular cycles and prolonged periods in the estrus phase [30]. Additionally, impaired progesterone responsiveness could contribute to endometrial dysfunction. There also has been reports that adenomyosis can lead to impaired endometrial receptivity, which is associated with a lack of adequate expression of adhesion molecules, reduced expression of implantation markers, and altered function of genes involved in embryonic development [4]. In addition, adenomyosis contributes to decreased fertility through disrupted estrous cycling, compromised ovarian follicle development, and reduced fertility from the decreased expression of the endometrial progesterone receptor and receptivity-related genes [30]. Another theory of pathogenesis of adenomyosis and its cause of infertility is proposed to be a dysregulation of inflammatory markers. Impaired implantation is thought to occur from the increased expression of inflammatory markers such as IL-1β and corticotropin-releasing hormones and interleukin-1β [31]. The dysregulation of HOXA, leukemia inhibitory factor, and matrix-metalloproteinase-2 inhibits decidualization along with reduced uterine receptivity due to decreased expression of integrin family proteins, cell adhesion receptors, and extracellular matrix protein enzymes (specifically osteopontin and interleukin-β3,1) [31]. The increase in inflammatory markers has been shown in increased local production of estrogen leading to a cycle of microtrauma and impaired tissue repair [5]. A recent study proposed retrograde uterine contractions during menstruation as an another theory of pathogenesis of adenomyosis and endometriosis [32]. In this meta-analysis, women with retrograde direction of uterine contractions were found to have higher prevalence of endometriosis and adenomyosis (RR, 8.63; 95% CI, 3.24–22.95) [32]. While there are many available theories, there is still much work that needs to be conducted to elucidate the exact pathogenesis of adenomyosis to understand the downstream effects on fertility.

## 5. Adenomyosis and Effect on Fertility

Decreased fertility in women with adenomyosis highlight the profound impact of the condition on reproductive outcome. Adenomyosis in women with infertility has been encountered more frequently due to improved diagnostic testing and an increased number of women seeking out fertility treatment [12]. In women with adenomyosis, abnormal levels of free radicals have been found in the uterine cavity, which can also negatively impact oocyte quality and inhibit embryo development and implantation, resulting in reduced pregnancy rates [30]. In a systematic review, pregnancy rates were reported to be lower in patients with adenomyosis when compared to women undergoing ART for different reasons, OR (odds ratio) 0.69 (95% confidence interval: 0.51–0.94), while higher miscarriage rates were noted, OR 2.17 (95% confidence interval: 1.25–3.79) [33]. The considerations to take when adenomyosis is present in an assisted reproductive technology (ART) setting are still not well understood and often overlooked [34]. It has been shown that adenomyosis negatively impacts in vitro fertilization (IVF) outcomes through reduced rates of implantation and pregnancy, increased risk of early pregnancy loss, and, as a result, a decrease in live birth rate [12,34]. Additionally, one study found a direct relationship between increased uterine size (uterus larger than that at 8 weeks gestation) and higher miscarriage rates in IVF [35]. In addressing patient concerns, it is critical to ensure fertility desires are understood as it is critical in discussing management options. This review details possible treatment options for patients when uterine preservation versus definitive treatment is desired.

## 6. Management of Adenomyosis

### 6.1. Patients Seeking Fertility

#### 6.1.1. Levonorgestrel Intrauterine Device (IUD)

Despite the symptoms of adenomyosis and impact on quality of life, there are currently no U.S. Food and Drug Administration-approved medical management options, particularly for younger women [17]. While there are other medical options being studied for use in adenomyosis such as GnRH agonists, the levonorgestrel IUD has been shown to be effective in the management of symptoms of adenomyosis. The IUD is a small device that is inserted into the uterus releasing progesterone to suppress menstruation as a possible option for symptomatic relief in patients with adenomyosis. In a study looking at the efficacy of IUD in the treatment of adenomyosis, there was a 56.2% retention rate with improvement in bleeding in 47.8% of patients [36]. The levonorgestrel system has been shown to also cause local atrophy of adenomyotic lesions leading to a decrease in overall burden [4]. There have been reports of increased expulsion, up to 17%, from the increased uterine size in patients with adenomyosis, but the exact expulsion rate has been difficult to fully understand from the lack of large population-based studies [37]. Additionally, in a study conducted to understand the rate of progression of adenomyosis, 21.3% of patients were noted to have progression of symptoms and/or US findings of disease within 1 year. In hormonally treated patients, 18.34% was shown to have progression while 30.77% of hormonally untreated patients had progression of disease [38]. For this reason, the IUD, being a removable system, may be a viable option for patients seeking fertility in the future.

#### 6.1.2. Hyperthermic Therapy Modalities

Radiofrequency ablation (RFA) refers to the broad class of therapies utilizing hyperthermic modalities for treatment of various conditions, and there has been interest in its effect at uterus sparing treatment of adenomyosis [39]. In this group of therapies, the treatment focuses on delivering heat energy to a focal lesion through high frequency energy of different sources. In these newly explored therapies, electrodes induce thermal ablation in the target lesion by focusing beams of energy at the desired point with minimal or no damage to the surrounding normal tissue. In radiofrequency ablation, high frequency alternating electrical energy is used, while in high-intensity focused ultrasound (HIFU) therapy, ultrasound energy is utilized. Additionally, US-guided percutaneous microwave ablation (PMWA) for adenomyosis has gained increasing attention in recent years due to better efficacy, higher efficiency, and fewer complications [31]. In one large study conducted with 15,123 patients, relief of dysmenorrhea was noted in 84.2% of patients undergoing HIFU, 89.7% of patients undergoing PMWA, and 89.2% of patients undergoing RFA [40]. In a separate systematic review, 50–94.7% of patients reported improvement in pain, 25–80% of patients reported improvement in bleeding [2]. The recurrence rate was noted to be about 9–19% [2]. Compared to other therapies such as uterine artery embolization, it has the advantage of less invasiveness, a lower incidence of severe complications, and no radiation exposure. It may be a therapy that could be used for patients who desire fertility. However, it is uncertain how this therapy affects future fertility given its novelty, so care should be given to patients who desire fertility.

#### 6.1.3. Adenomyomectomy

Surgical excision of adenomyosis can be performed for those who desire uterine preservation. However, the contraindications and future fertility recommendations are mixed depending on the pervasiveness of the adenomyosis. Subsequent pregnancies may have high miscarriage rates, and silent uterine ruptures may occur during mid-term pregnancy due to thinning of the uterine walls [41,42]. Even if there is localized adenomyosis, an adenomyomectomy can be technically challenging due to differentiation of the tissue planes and closure of the myometrium following excision. Additionally, similar operative and postoperative risks can be seen with myomectomy. In a systematic review looking at uterine-conserving surgical techniques, there was overall improvement in uterine volume, pain, and bleeding in patients undergoing surgery, showing the effectiveness of the treatment [43]. For patients considering adenomyomectomy, utilizing multiple imaging modalities including MRI can be helpful for surgical planning. Considerations should be given to the classification of the lesion (diffuse versus focal), depth of the lesion, and the amount of junctional zone involvement as these will all impact the surgical approach. Focal and localized adenomyosis lesions can be treated laparoscopically; however, diffuse adenomyosis must be treated by laparotomy or preferably by laparoscopically assisted laparotomy [41]. Open surgery has been considered safer than laparoscopy for diffuse adenomyosis in that it can thoroughly excise the lesions to prevent recurrence because of the ability to palpate even small lesions and to properly reconstruct the defect created by the surgery to prevent uterine rupture in subsequent pregnancies [32]. Patients undergoing adenomyomectomy should be counseled about the uncertain impact of these procedures on fertility and pregnancy. Significantly increased risk of uterine rupture during the second and third trimesters of pregnancy have been seen, with rates of rupture ranging from 0.8% to 6% [41]. There is minimal literature evaluating the long-term impacts on the quality of life or recurrence in those with adenomyomectomy [44]. In a prior review, some techniques for decreasing hemorrhage during myomectomy were described, and given the nature of the surgery, similar principles can be applied to adenomyomectomy. Some interventions that were shown to be useful include intramyometrial vasopressin (decreases blood loss by −245.87 mL, 95% confidence interval of −434.58 mL to −57.16 mL), vaginal misoprostol/dinoprostone (−97.88 mL with a 95% confidence interval of −125.52 mL to −70.24 mL), tourniquet around the cervix (−240.70 mL with a 95% confidence interval of −359.61 mL to −121.79 mL), and intraoperative IV tranexamic acid (−243 mL with a 95% confidence interval of −460.02 mL to −25.98) [45].

#### 6.1.4. Considerations for Surgical Management

There is no consensus to date on the best candidates for the surgical treatment of adenomyosis. A systematic review showed that there was no statistically significant difference in pregnancy, miscarriage, and live birth rates following excisional vs. conservative treatment [46]. There was no difference noted in pregnancy outcomes in patients with diffuse or localized disease [46]. In one study, age ≤ 39 was used as a determinant for surgical management, and it found that the change in pregnancy following surgery was 41.3% compared to 3.7% in those who were 40 years and older [47]. They found that the highest increased pregnancy following surgery was seen in patients who had previously failed IVF cycles and were ≤39 years old (60.8%) [47]. The authors for this study used a multivariate regression model that showed that posterior wall involvement was significantly different in patients able to achieve pregnancy after surgery (*p* = 0.0015) and had a history of IVF treatment (*p* = 0.038) [47]. These studies show that excisional procedures should be considered for younger patients desiring fertility with significant pain from disease or multiple years of infertility with failed IVF treatment cycles [46]. This should be balanced with the risks associated with surgery including the risk of uterine rupture during pregnancy, intrauterine and extrauterine adhesion formation, and decline in ovarian function [46].

Finally, while endometriosis and adenomyosis can be found concomitantly in many patients, this review focuses on treatment options directed to adenomyosis. However, the high prevalence of both disorders should be a consideration for surgical planning given that the extent and involvement of the surgery could change. Additionally, given the risks associated with extensive surgical management for both disorders, there should be thorough discussions about the patient’s goals related to surgical management and fertility.

### 6.2. Considerations for ART

#### 6.2.1. Discussion on Ovarian Stimulation

There is now more data demonstrating the negative impact adenomyosis has on fertility which has been also demonstrated with negative IVF outcomes in patients with adenomyosis. Recently, there has been more attention paid in understanding different ways of overcoming these negative impacts. A protocol that has been more extensively studied is GnRH agonist (GnRHa) pretreatment. GnRHa is thought to have an antiproliferative effect on adenomyotic tissue, helping to decrease inflammation and to induce apoptosis [12]. Some studies show that a 3-month pretreatment, as compared to a long GnRHa protocol, seems to have more favorable IVF outcomes. Smaller case studies have shown increased oocytes retrieved with these protocols despite unfavorable AMH values [45]. While long-term GnRHa has been shown to be effective in symptomatic control and control of adenomyosis lesions can cause uterine size reductions, it is a costly treatment and can have negative impacts on bone density with prolonged use [48]. Additionally, in patients who are awaiting to undergo IVF cycles, there is very little research on which medications are more effective at controlling symptoms while having limited negative impacts on the cycle. In one study, letrozole was studied as a low-cost alternative to help control adenomyosis symptoms in patients awaiting IVF cycles compared to GnRHa. This study showed no difference in treatment with letrozole compared to GnRHa in these patients [49]. For this reason, letrozole may be an alternative to suppress symptoms of adenomyosis prior to IVF cycles in patients while GnRHa pretreatment seems to have positive effects on cycle outcomes for adenomyosis patients.

#### 6.2.2. Suppression with Embryo Transfer

Many studies have demonstrated a decrease in live birth rate and increased miscarriage rate for patients with adenomyosis even with ART. There have been reports of increased risk of recurrent implantation failure in these patients possibly from dysregulation of myometrial architecture and function, chronic inflammation with increased local oxygen species, and altered endometrial function [50]. For this reason, there are reports favoring frozen embryo transfers with pretreatment with GnRH agonists [3]. The clinical pregnancy rate for patients with pretreatment along with a frozen embryo transfer was 39.5% compared to 30.5% in patients who received pretreatment but proceeded with a fresh embryo transfer; the *p* value was not significant [3]. Additionally, another study showed pretreatment with 3 months of GnRHa prior to the frozen-embryo transfer helped significantly increase the live birth rate (46.7% vs. 24.8%, *p* = 0.009) and significantly decrease the miscarriage rate (12.5% vs. 37.2%) when compared to no pretreatment for patients with adenomyosis, so a significant decrease in uterine volume or statistically significant increase in the clinical pregnancy rate (53.3% vs. 39.4%, *p* = 0.044) was noted in the study patients [35]. From the available data, GnRHa pretreatment seems to help increase pregnancy outcomes in patients with adenomyosis along with frozen transfer to allow for suppression of adenomyotic lesions prior to the transfer.

### 6.3. Patients Not Seeking Fertility

#### 6.3.1. Uterine Artery Embolism (UAE)

Uterine artery embolism is an angiographic procedure that is conducted for injecting embolic agents into the uterine arteries to cause ischemic atrophy of uterine pathology such as adenomyosis and leiomyoma. There is a more aggressive introduction of embolic agents to cause complete stasis of blood flow for the treatment of adenomyosis [51]. While the data for UAE for leiomyoma are more abundant and promising, the data about efficacy in adenomyosis are more limited. In the past, there was questionable efficacy of UAE for the treatment of adenomyosis, with only 55% of patients reporting any benefit from the procedure at 2 years [51]. More recently, smaller studies have shown more significant improvement—an 82% improvement in symptomatic adenomyosis—however, data are limited [52,53]. As of now, there is a lack of good evidence on the effect of UAE on fertility in patients with adenomyosis. While there are patients who go on to have pregnancies without complications following UAE for leiomyomas, given the difference in technique, there is still research that is needed for understanding the effects [51]. Because of this lack of evidence for safety in subsequent pregnancies, patients should be counseled about the risks prior to undergoing this procedure.

#### 6.3.2. Endometrial Ablation (EA)

Endometrial ablation is a minimally invasive surgical procedure that can induce amenorrhea or menstrual bleeding volume reduction by directly destroying the basal layer of the endometrium and its deep junction zone. This is an outpatient procedure that can be offered to people with adenomyosis who desire uterine preservation but do not have intentions for continued fertility. Patients with plans for future fertility are not appropriate for EA because pregnancies after EA are associated with increased rates of morbidly adherent placenta [54]. A report of repeat EA has demonstrated improved menorrhagia and patient satisfaction; however, it has shown increased inherent potential for tissue injury [55]. Significant reduction in bleeding in 93% and 67.5% of patients was achieved at 6 months and 3 years, respectively [56]. Dysmenorrhea related to adenomyosis improved in 60.6% and 51.5% of patients at 6 months and 3 years, respectively [56]. Endometrial ablation works at the layer of the endometrium, so deep layers of the myometrium are unaffected. The efficacy of this therapy can decrease over time and recurrence of adenomyosis-related symptoms may require further therapy [56]. The rate of hysterectomy at the 3-year time point was 18% due to symptom burden or recurrence [56]. Despite this, it remains a viable option for symptomatic patients seeking treatment for adenomyosis.

#### 6.3.3. Hysterectomy

For those who desire definitive treatment, long-term therapies remain limited beyond hysterectomy [57]. The crude percentage of people who undergo hysterectomy overall in the US is 17.2% [58]. Hysterectomy is considered the most definitive treatment for adenomyosis in premenopausal and postmenopausal women. Adenomyosis is often found on tissue diagnosis following hysterectomy. When evaluating those who had a hysterectomy for all types of reasons, 59.3% had histologically proven adenomyosis [59]. Total hysterectomy can be performed vaginally, laparoscopically, robotically, or open [60]. Postoperatively, 42.0% of laparoscopic hysterectomies performed had histologically proven adenomyosis [59]. In vaginal hysterectomies, adenomyosis was found in 12% of pathology. Hysterectomy, compared to conservative management, is an invasive surgery that requires longer operative time and recovery time. Medical and surgical comorbidities may impact surgical outcomes and limit patients from receiving a hysterectomy for adenomyosis. Important considerations for hysterectomies in the setting of adenomyosis include possible increased risk due to uterine size and vascularization of the uterus. These attributes of adenomyosis can increase the risk of blood loss during surgery. A meta-analysis published in 2014 reports that the overall rate of urinary tract injury due to laparoscopic hysterectomy was 0.73%; bladder injury rates ranged from 0.05% to 0.66% and ureteral injury rates ranged from 0.02% to 0.4% [61]. Despite this, hysterectomy remains the standard of care for adenomyosis and is considered the most therapeutic option and standard of care for adenomyosis treatment as it does not have potential to recur if the uterus is removed [41].

## 7. Discussion

Adenomyosis has become a more prevalent diagnosis and there has been more research into the effects on fertility. Additionally, clinicians have become more cognizant of fertility desires of patients presenting with symptomatic adenomyosis. Through this literature review, multiple treatment options were presented for patients who desire fertility preservation versus those that want definitive treatment. Despite the increased research efforts into adenomyosis, there is still a lack of defined diagnostic criteria, and the gold standard remains pathologic diagnosis with hysterectomy. Additionally, there is limited research into the pathophysiology of adenomyosis which is important in understanding the extent of effects on fertility. As women are delaying childbearing, there will be an increased prevalence of adenomyosis in patients undergoing fertility treatments. There is still no clear consensus on specific patients who would benefit from surgery. For this reason, there still needs to be a better understanding of the best ways to maximize oocyte quality, implantation, and live birth rate in patients with adenomyosis.

## Figures and Tables

**Table 1:** Ultrasound classification of adenomyosis based on adenomyosis involvement.

Adenomyosis Classification	Definition of Lesion	Location of Lesion
Diffuse Adenomyosis	Ill-defined	Global myometrial and uterine wall thickening with junctional zone thickening
Focal Adenomyosis	Usually ill-defined but can be defined	Localized lesion in the outer myometrium with possible lesions in the junctional zone
Adenomyoma	Well-defined	Found throughout and is usually categorized by size
